# Phase II randomised trial of chemoradiotherapy with FOLFOX4 or cisplatin plus fluorouracil in oesophageal cancer

**DOI:** 10.1038/sj.bjc.6605943

**Published:** 2010-10-12

**Authors:** T Conroy, Y Yataghène, P L Etienne, P Michel, H Senellart, J L Raoul, L Mineur, M Rives, X Mirabel, B Lamezec, E Rio, E Le Prisé, D Peiffert, A Adenis

**Affiliations:** 1 Centre Alexis Vautrin, Department of Medical Oncology, Nancy University, 6 avenue de Bourgogne, 54511 Vandœuvre-lès-Nancy Cedex 54511, France; 2 Sanofi-aventis, 9 boulevard Romain Rolland, 75159 Paris Cedex 14, France; 3 Clinique Armoricaine de radiologie, 21 rue du Vieux Séminaire, Saint-Brieuc Cedex 22015, France; 4 Unité d’oncologie digestive, Service d’hépato-gastroentérologie Hôpital Charles Nicolle – CHU de Rouen, 1 rue de Germont, Rouen Cedex, 76031, France; 5 Service de radiothérapie, Centre René Gauducheau, Boulevard Jean Monot, Nantes 44805, France; 6 Département d’oncologie médicale, Centre Eugène Marquis, CS 44229, Rennes 1 University, Rennes Cedex 35042, France; 7 Service de Radiothérapie, Clinique Sainte Catherine, 1750 chemin Lavarin, Avignon 84000, France; 8 Service de radiothérapie, Institut Claudius Regaud, 20-24 rue du Pont Saint Pierre, Toulouse Cedex 31052, France; 9 Service de radiothérapie, Centre Oscar Lambret, 3 rue Frédéric Combemale, Lille Cedex 59020, France; 10 Service de radiothérapie, Centre Eugène Marquis, CS 44229, Rennes Cedex 35042, France; 11 Service de cancérologie digestive et urologique, Centre Oscar Lambret, 3 rue Frédéric Combemale, Lille Cedex 59020, France

**Keywords:** oesophageal cancer, chemoradiotherapy, oxaliplatin

## Abstract

**Background:**

Concurrent chemoradiotherapy is a valuable treatment option for localised oesophageal cancer (EC), but improvement is still needed. A randomised phase II trial was initiated to assess the feasibility and efficacy in terms of the endoscopic complete response rate (ECRR) of radiotherapy with oxaliplatin, leucovorin and fluorouracil (FOLFOX4) or cisplatin/fluorouracil.

**Methods:**

Patients with unresectable EC (any T, any N, M0 or M1a), or medically unfit for surgery, were randomly assigned to receive either six cycles (three concomitant and three post-radiotherapy) of FOLFOX4 (arm A) or four cycles (two concomitant and two post-radiotherapy) of cisplatin/fluorouracil (arm B) along with radiotherapy 50 Gy in both arms. Responses were reviewed by independent experts.

**Results:**

A total of 97 patients were randomised (arm A/B, 53/44) and 95 were assessable. The majority had squamous cell carcinoma (82% arm A/B, 42/38). Chemoradiotherapy was completed in 74 and 66%. The ECRR was 45 and 29% in arms A and B, respectively. Median times to progression were 15.2 and 9.2 months and the median overall survival was 22.7 and 15.1 months in arms A and B, respectively.

**Conclusion:**

Chemoradiotherapy with FOLFOX4, a well-tolerated and convenient combination with promising efficacy, is now being tested in a phase III trial.

Although significant advances in the staging and treatment of patients with oesophageal cancer (EC) have recently been seen, this malignancy is still lethal for the majority of patients (American Cancer Society, 2008). Chemoradiation was established as one of the viable options for therapy of patients with localised EC following the results from the landmark trial of the Radiation Therapy Oncology Group (RTOG) 85-01 trial, showing a significant survival advantage of concurrent chemoradiation over radiation alone (Al-Sarraf *et al*, 1997; Cooper *et al*, 1999).

However, the local failure rate was 45% in the chemoradiation arm (Al-Sarraf *et al*, 1997). It is worth to note that a majority of squamous cell carcinomas (80%) were included in the RTOG 85-01. Moreover, the small cohort of adenocarcinomas treated with chemoradiation achieved a lower 5-year survival rate as compared with squamous cell (13 *vs* 21%) (Cooper *et al*, 1999). In addition, 20% of patients who received chemoradiation experienced life-threatening side effects, and chemotherapy could not be completed in >40% of the patients (Al-Sarraf *et al*, 1997). Therefore, safer and more effective non-operative treatments are expected.

The FOLFOX4 regimen is a combination of oxaliplatin, fluorouracil and leucovorin, which has demonstrated efficacy and good tolerability in colorectal cancer (De Gramont *et al*, 2000; André *et al*, 2004). A similar regimen showed a good response rate (40%) and a 1-year survival rate of 31% in advanced EC (Mauer *et al*, 2005). In 2004, we performed a phase I trial to assess the dose-limiting toxicity of the combination of radiotherapy with oxaliplatin, fluorouracil and leucovorin every 2 weeks (Conroy *et al*, 2008). The recommended doses for phase II studies of fluorouracil and oxaliplatin with concurrent radiation were those of the FOLFOX4 regimen (detailed below). Afterwards, we started a randomised phase II–III study comparing our experimental chemoradiation regimen with FOLFOX4 to the standard cisplatin/fluorouracil chemoradiation combination in patients with EC.

## Patients and methods

### Design

This was a multicentre, randomised, open-label, phase II study to test the feasibility (completion of full treatment) of the FOLFOX4 regimen *vs* the cisplatin/fluorouracil regimen in EC patients. Patients were stratified for study centre and histological type. The primary end points were the completion of full treatment and endoscopic complete response rate (ECRR). Treatments were considered as fully completed if patients received full doses of radiotherapy and all cycles of chemotherapy (six cycles of FOLFOX4 or four cycles of cisplatin/fluorouracil). An independent data monitoring committee (IDMC) was set up to review the ECRR, safety and other issues related to the conduct of the study.

The study was conducted in accordance with the Declaration of Helsinki, Good Clinical Practice guidelines, and legal requirements. The protocol was approved by the Ethical Committee of Lorraine. Written informed consent was obtained from all patients.

Three conditions needed to be fulfilled before initiating the phase III part of the study: fast accrual rate (88 patients within 18 months), completion of full treatment in >60% of patients in the experimental arm, and ECRR of FOLFOX4 equal or superior to ECRR of cisplatin/fluorouracil.

### Patient eligibility

Patients had to have histologically proven adenocarcinoma or squamous cell or adenosquamous EC (any T, N0 or N1, M0 or M1a) and previously untreated. Patients with technically unresectable cancer or those with surgical contraindications and those who refused to undergo surgery were eligible. Additional inclusion criteria were age ⩾18 years, Eastern Cooperative Oncology Group (ECOG) Performance Status (PS) ⩽2, peripheral neuropathy ⩽NCI-CTC grade 1, sufficient caloric intake, life expectancy ⩾3 months, and adequate bone marrow reserve, normal renal and liver functions. Non-inclusion criteria were multiple ECs, weight loss >20% normal body weight, previous radiotherapy, invasion of the tracheo-bronchial tree, previous myocardial infarction, symptomatic arteritis and other serious illness or medical conditions.

Patients were randomised in a 1 : 1 ratio. A central stratified block randomisation procedure was used to balance prognostic factors between treatment arms and to minimise the predictability of treatment allocation in this open label study. Investigator centre and histology type were used as strata.

Each centre was attributed a fixed number of blocks, which were allocated according to histology type. The SAS function ‘RANUNI’ with an arbitrary ‘SEED’ value was used to generate a random sequence of permutations, which was used to produce a list of patient randomisation numbers. The numbers were then allocated to each centre and strata in equal frequencies for each treatment arm.

### Treatment

All patients were scheduled to receive concomitant chemoradiotherapy followed by chemotherapy alone. The first fraction of radiotherapy and the first cycle of chemotherapy began on the same day.

#### Radiation

The radiation dose regimen was the same in both arms. Megavoltage >6 MV was used with 3–4 beams and a total dose of 50 Gy at the intersection of all fields and to lymph nodes if any; 2 Gy per fraction, five fractions per week were delivered. All fields were used every day and the maximum dose to the spinal cord was 40 Gy. Computerised imaging was used to define the target volumes.

The target volume included the primary tumour, that is, the gross tumour volume, distal and proximal margins of 3–5 cm and lateral margins of 2 cm at mediastinal interface (PTV). The accepted PTV coverage had to respect the constraints of the ICRU report, between 95 and 107% of the prescribed dose. For upper third primaries, the subclavicular lymph nodes were included. For middle third tumours, the right and left retro-clavicular regions were included in the initial target volume when the upper limit of the tumour exceeded the carena. For lower third cancers, the irradiated field included the coeliac lymph nodes. The choice of technique (number and orientation of the beams) resulted from the analysis of the lung dose–volume histogram and CTV. Whenever possible, the following constraints were respected: lung dose (volume of the two lungs – PTV) receiving >20 Gy (V20) <20% of the total lung volume. Control portal images were to be performed for each field at the start and at each ballistic change.

#### Chemotherapy

**Figure 1:**
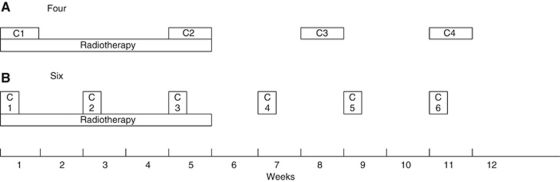
Treatment schedules. (**A**) Cisplatin/fluorouracil: four cycles of 4 days. (**B**) FOLFOX4: six cycles of 2 days.

Patients randomised to arm A received, every 2 weeks, six cycles of FOLFOX4, the three first cycles concomitant with radiotherapy. The FOLFOX4 regimen (doses in mg m^−2^) was oxaliplatin 85 on day 1 and leucovorin 200, both as a 2-h intravenous infusion then bolus fluorouracil 400 followed by fluorouracil 600 in 22-h infusions on days 1 and 2. Treatment with FOLFOX4 continued after the end of radiotherapy for three cycles (Figure 1). Patients randomised to arm B received two cycles of cisplatin/fluorouracil on weeks 1 and 5 during radiotherapy and two other cycles after the end of radiotherapy on weeks 8 and 11. The chemotherapy regimen included cisplatin 75 mg m^−2^ on day 1 with standard hydration followed by fluorouracil 1000 mg m^−2^ day^−1^ i.v. by continuous infusion from day 1 to day 4 of each cycle (Al-Sarraf *et al*, 1997; Cooper *et al*, 1999). Both chemotherapy regimens were stopped in case of disease progression, unacceptable toxicity or patient refusal. Dose reductions and delays for chemotherapy and interruptions for radiotherapy were planned according to toxicities. If interruption of either radiotherapy or chemotherapy lasted >2 weeks, the patient was considered off study and was followed for safety. Appropriate antiemetics were prescribed. Hematopoietic growth factors and oesophageal dilatation were not permitted during treatment.

### Pretreatment evaluation and assessments

All patients underwent a complete history and physical examination, ECG, biological and CBC before entry, then weekly CBC and every 2 weeks, physical examination and safety evaluation of the treatment along with biochemistry. Tumour assessments were performed with CT scan, barium swallow, oesophagoscopy and biopsies, bronchoscopy, and endoscopic ultrasonography (EUS) when feasible. Tumours with tight malignant oesophageal strictures that preclude passage of the echoendoscope were considered at least T3 stage and were evaluated with CT scan. In both arms, tumour evaluations were repeated at week 15 and every 6 months in complete responders until disease progression. If the response was considered as partial at the first assessment or in case of any doubt on endoscopic response (ulceration or stenosis), a second evaluation with CT scan, endoscopic examination and biopsies were to be performed within 2 months.

### Evaluations

#### Full treatment

Patients were considered as having completed the treatment if they had received six cycles of FOLFOX4 or four cycles of cisplatin/fluorouracil.

#### Tumour response

Patients had to have received 50 Gy and a minimum of three cycles of FOLFOX4 or two cycles of the cisplatin/fluorouracil to be considered evaluable for response. Tumour responses were assessed during week 15 according to Response Evaluation Criteria in Solid Tumours (RECIST) Guidelines (Therasse *et al*, 2000). The primary tumour was assessed on CT scan with measure of the vertical length and maximal thickness of the oesophageal wall on transverse plane. Endoscopic complete response (CR) was defined as the complete disappearance of any tumour, ulceration or stenosis with no new lesion (all endoscopic pictures and reports had to be available), observation of the entire oesophagus as defined by Tahara *et al* (2005), but also no CT-scan progression. Biopsies were not mandatory. Assessment of endoscopic CR was performed by the IDMC on the population evaluable for response, that is, those who received concomitant chemoradiotherapy. An intent-to-treat analysis (on all randomised patients) was planned for all other end points. Progression-free survival (PFS) was defined as the date from randomisation to tumour progression or death. Overall survival was measured from date of study entry to date of death.

#### Toxicity

All patients who had received at least one dose of chemotherapy and/or one fraction of radiotherapy were considered evaluable for safety. Toxicities were recorded according to the NCI-CTC version 3 (http://ctep.cancer.gov/protocoldevelopment/electronic_applications/docs/ctcaev3.pdf).

### Statistical analysis

#### Determination of sample size

A sample size of 40 patients in each arm was designed to achieve at least 85% power, to observe 40% endoscopic CR, and to exclude a lower limit of confidence interval (CI) of 20% CR. A 10% adjustment for drop-outs resulted in a sample size of 88 patients. A two-sided, binomial hypothesis test with a target significance level of 0.05 was used.

#### Descriptive statistics

The per-protocol population corresponds to all eligible patients in whom endoscopic response may be evaluated. The Kaplan–Meier method was used to estimate survival rates. Hazard ratios and 95% CIs were estimated from Cox proportional hazards models adjusted for the stratification factor used in the randomisation. Analyses were generated using SAS version 8.2 or higher (SAS Institute, Cary, NC, USA).

## Results

**Figure 2:**
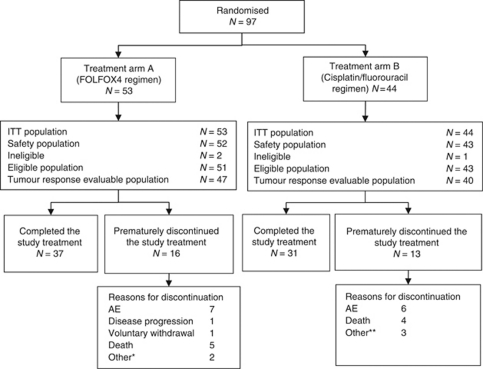
CONSORT diagram. ^*^One patient had hepatic and pulmonary metastases and one patient had oesophageal prothesis. ^**^One patient had ischaemia on myocardial scintigraphy, one patient was not compliant and one patient did not start chemotherapy because of one SAE.

From October 2004 to December 2005, 97 patients were randomised in 21 centres (Figure 2): 53 patients were included in the FOLFOX4 regimen (arm A) and 44 patients in the cisplatin/fluorouracil regimen (arm B). Two ineligible patients (one in each arm) did not receive any treatment (one metastatic, one myocardial ischaemia before treatment). Another patient in arm A was considered ineligible (*in situ* carcinoma), but was included in the safety population for response. A total of 95 patients (97.9%) were included in the safety population (arm A/B, 52/43). Four other patients in arm A and three patients in arm B were not evaluable for tumour response due to oesophageal prothesis, no chemotherapy or incomplete chemotherapy and/or dose of radiotherapy <45 Gy.

### Patient characteristics

Both arms were similar for demographic and baseline characteristics, although slightly more patients had a baseline ECOG PS of 0 in arm A (67.9%) as compared with arm B (54.5%). The majority of patients had squamous cell carcinoma and stage III disease located in the middle thoracic oesophagus (Table 1). One third of patients (35/97, 36%) had cervical or upper third thoracic tumours. The proportion of cT3 tumours was similar in both arms (arms A/B: 79.2%/81.2%).

### Completion of treatment

A total of 68 patients (70.1%) completed the treatment. Premature discontinuation of treatment was due to adverse events (AEs; 13.2%/13.6% in arms A/B) or death (9.4%/9.1%, in arms A/B; Figure 2). In the ITT population, 37 patients (69.8%) in arm A and 31 patients (70.4%) in arm B completed the full treatment, including all cycles of chemotherapy. In arm A, dose reductions were observed in 13 out of 52 patients (25%) for oxaliplatin and in 19 out of 52 patients (36.5%) for fluorouracil. In arm B, 9 out of 43 patients (20.9%) had cisplatin dose reduction and 14 out of 43 (32.6%) had fluorouracil dose reduction. The majority of dose reductions were due to neutropenia or thrombocytopenia. Almost all patients (>97%) in both treatment arms completed the 5 weeks of radiotherapy. The median radiotherapy dose received was 50 Gy (range: 28–50) in arm A and 50 Gy (range: 14–50) in arm B. Six patients (11.5%) in arm A and three patients (7%) in arm B had an AE leading to temporary discontinuation of radiotherapy.

### Response

According to the IDMC, an endoscopic CR was reported in 21 out of 47 patients (44.7% 95% CI: 30.2%, 59.9%) in arm A and in 12 out of 40 patients (30% 95% CI: 15.8%, 44.2%) in arm B (Table 2). According to the investigators, CR was observed in 20 patients (42.6% 95% CI: 28.2%, 57.8%) in arm A and 18 patients (43.9% 95% CI: 28.5%, 60.3%) in arm B. Reasons for discrepancies between investigator assessments and IDMC (CR downgraded to no CR) were persistence of an oesophageal ulceration (six patients) and persistent impassable stenosis (three patients). Two patients assessed as CR by the investigator (one in each arm) were considered as nonevaluable for response (according to the protocol) by the IDMC because only one or two cycles of chemotherapy were administered. One patient in the FOLFOX arm claimed as CR by the investigator was considered as nonevaluable by the IDMC members because of an oesophageal prosthesis. The IDMC also reclassified two PR in CR because of a further normal endoscopy with negative biopsies and normal CT scan.

### Survival

**Figure 3:**
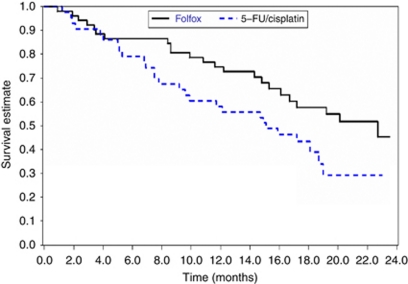
Overall survival (ITT population).

The median follow-up was 15 months. Median survival time was 22.7 months (95% CI: 16.1% -not reached) in arm A and 15.1 months (95% CI: 9.6%, 19.0%) in arm B. At 1 year, the survival rate was 75% in arm A and 58% in arm B. At 3 years, the survival rates were 45% (95% CI: 28%, 63%) in arm A and 29% (95% CI: 13%, 46%) in arm B (Figure 3).

### PFS

The median PFS was 15.2 months (95% CI: 10.4%, 21.7%) in arm A and 9.2 months (95% CI: 6.9%, 14.5%) in arm B.

### Safety

The most frequently reported AEs (all grades) were nausea and neutropenia. Treatment-related paresthesias occurred in 24 patients (46.2%) in arm A and 2 patients (4.7%) in arm B.

A total of 31 patients (59.6%) in arm A and 27 patients (62.8%) in arm B had grade 3 or 4 related AE (Table 3). The most frequently reported grade 3–4 related AEs were neutropenia and dysphagia. No grade 3–4 diarrhoea occurred. A total of 32 patients (61.5%) in arm A and 24 patients (55.8%) in arm B reported serious AE (SAE). The most frequently reported SAEs were dysphagia and febrile neutropenia.

Five deaths were considered possibly related to the study treatment: one in arm A (pneumopathy and denutrition in a context of progressive disease), and four in arm B (pancytopenia, pulmonary infection/grade 4 neutropenia/dehydration/stroke, cardiac ischaemia, infection/neutropenia). All were male and had squamous cell carcinoma and no obvious risk factor except ECOG PS 2 in one patient and age over 70 in another patient.

## Discussion

This FOLFOX4–radiation combination achieved an endoscopic CR in 44.7% of patients with acceptable toxicity and one possibly treatment-related death. The control arm with cisplatin–fluorouracil did apparently worse as endoscopic CR was observed in only 29.3% of patients and four toxic deaths were noted. The endoscopic CR was chosen as the primary end point for the phase II part of this study for convenience and because it was demonstrated to be a good surrogate end point for overall survival (Brown *et al*, 2004; Kalha *et al*, 2004). The other reason was that CT scan and EUS are notoriously unreliable in the restaging after chemoradiation (Kalha *et al*, 2004; Westerterp *et al*, 2005). Persistence of a residual thickening of the oesophageal wall on CT scan is not incompatible with the subsequent finding of a histological CR as demonstrated in neoadjuvant studies (Westerterp *et al*, 2005). This low accuracy of imaging techniques is related to the difficulty in differentiating viable tumour from inflammatory changes or scar tissue (Kalha *et al*, 2004; Westerterp *et al*, 2005). FDG-PET scan showed some promises for restaging tumours, but it was not widely available at the time this study was designed.

RECIST guidelines do not refer to endoscopy CR criteria in much detail (Therasse *et al*, 2000). No studies on chemoradiotherapy for localised EC have precisely defined CR criteria for the primary tumour as outlined by Tahara *et al* (2005). In our study, endoscopic CR was precisely defined upon endoscopic observation of the entire oesophagus as disappearance of the tumour lesion, no ulceration, no budding, no new lesion on endoscopy and no local progression on CT scan. Rebiopsy after chemoradiation was not mandatory in this trial because at the time it was designed, several studies, performed in the neoadjuvant setting, have shown a high false negative rate of biopsies in this setting (Poplin *et al*, 1987; Vogel *et al*, 1995; Adelstein *et al*, 1997; Bedenne *et al*, 1998; Triboulet *et al*, 1998). Endoscopic biopsy samples of superficial mucosa cannot identify residual tumour deeper within the oesophageal wall and limits its effectiveness. This has been subsequently confirmed in larger series (Schneider *et al*, 2008; Sarkaria *et al*, 2009).

The present study also showed that chemoradiotherapy with FOLFOX4 and 50 Gy is feasible in EC as ∼70% of patients completed the full treatment in the both arms. Acute toxicity was acceptable with the combination of FOLFOX4-radiation with only one related death compared with four deaths in the control arm. As compared with cisplatin, the administration of oxaliplatin is convenient and does not require planned hydration and considerably shortens the duration of outpatient visits. Other groups showed that different oxaliplatin-based regimens with concomitant radiotherapy provide promising activity with good tolerance (Khushalani *et al*, 2002; O’Connor *et al*, 2007; Leichman *et al*, 2009).

Median survival in the cisplatin–fluorouracil group was 15.1 months and is comparable to those obtain in the RTOG 85-01 study (14.1 months) and in the RTOG 94-05 trial (18.1 months in the 50.4 Gy arm; Al-Sarraf *et al*, 1997; Cooper *et al*, 1999; Minsky *et al*, 2002). In the FOLFOX4 group, the median survival was 22.7 months. Noteworthy, this rather good median survival was obtained while M1a staged patients were allowed to enter the study, contrary to the RTOG 94-05 study. This difference of 6–7 months in median PFS and survival in our trial appears promising.

This study has obvious limitations, including the imbalanced number of patients between both arms. Although no randomisation errors were identified during the study, more patients were randomised in arm A than in arm B. Actually, 21 active centres started at least one randomisation block and some of them were not attributed in totality to patients and, by chance, more allocations to the experimental arm were made in these noncompleted blocks. Few patients were ineligible, but comparable numbers of patients were evaluable for response and safety.

Both groups of patients were well balanced for the main survival prognostic factors, which were relevant at the onset of the trial, that is, stage, tumour length <50 mm and weight loss <10% (Coia *et al*, 2000; Thomas *et al*, 2004; Di Fiore *et al*, 2007). In other series, ECOG PS score 1 or 2 and adenocarcinoma type were identified as independent predictive factors of poor outcome (Gill *et al*, 1992; Cooper *et al*, 1999).

## Conclusion

Combination of FOLFOX4 and 50 Gy radiation showed a good efficacy/safety balance and achieved promising results. As the preplanned objectives of our study were fulfilled, a phase III study comparing the same regimens was started with PFS as primary end point.

This on-going study with other trials testing FOLFOX will define the place of the chemoradiotherapy with FOLFOX in the armamentarium of EC treatments.

## Figures and Tables

**Table 1:** Baseline characteristics (ITT population)

	**Arm A FOLFOX4 (*N*=53)**	**Arm B cisplatin/ fluorouracil (*N*=44)**
*Gender (n (%))*		
Male	45 (84.9%)	38 (86.4%)
		
*Age (years)*		
Median	59.0	58.0
Range	39–81	41–80
		
*ECOG PS at baseline (n (%))*		
0	36 (67.9%)	24 (54.5%)
1	17 (32.1%)	18 (40.9%)
2	0	2 (4.5%)
		
*Histological type (n (%))*		
Adenocarcinoma	11 (20.8%)	6 (13.6%)
Squamous cell carcinoma	42 (79.2%)	38 (86.4%)
		
*TNM classification (n (%))*		
Stage IIA	13 (24.5%)	15 (34.1%)
Stage IIB	6 (11.3%)	2 (4.5%)
Stage III	30 (56.6%)	24 (54.5%)
Stage IVA	4 (7.5%)	3 (6.8%)
		
*Location of primary tumour (n (%))*		
Cervical	6 (11.3%)	1 (2.3%)
Upper thoracic	14 (26.4%)	14 (31.8%)
Middle thoracic	21 (39.6%)	22 (50.0%)
Lower thoracic	14 (26.4%)	9 (20.5%)
		
*Inoperability (n (%))*		
Carcinologic reason	31 (58.5%)	25 (26.8%)
Therapeutic/patient choice	18 (34.0%)	12 (27.3%)
		
*Weight loss*		
<10%	32/52 (61.6%)	26/43 (60.5%)
⩾10%	20/52 (37.4%)	16/43 (37.2%)
Median tumour length (mm) (range)	50 (15–200)^a^	50 (23–130)

Abbreviations: ECOG=Eastern Cooperative Oncology Group; FOLFOX=oxaliplatin, fluorouracil and leucovorin; ITT=intent to treat; PS=performance status.

a Unavailable for one patient.

**Table 2:** Tumour response

	**FOLFOX arm A (*N*=47)**		**5FU-cisplatin arm B (*N*=40)**	
	***n* (%)**	**(95% CI) (exact)**	***n*** **(%)**	**(95% CI) (exact)**
*Complete endoscopic response rate assessed by the IDMC*				
*CR*	21 (44.7)	(30.2–59.9)	12 (30)	(15.8–44.2)
Squamous cell	17 (40.5)	(25.6–55.3)	10 (26.3)	(12.3–40.3)
Adenocarcinoma	4 (36.4)	(10.9–69.2)	2 (33.3)	(4.3–77.7)
No CR	18 (38.3)	(24.5–53.6)	21 (51.2)	(35.1–67.1)
Not evaluable	2 (4.3)	(0.5–14.5)	4 (10)	(0.7–19.3)
Not assessed	6 (12.8)	—	5 (12.2)	—
				
*Overall response rate assessed by the investigator (Tumour response evaluable population)–RECIST criteria*				
CR	20 (42.6)	(28.3–57.8)	18 (43.9)	(28.5–60.3)
PR	17 (36.2)	(22.7–51.5)	10 (24.4)	(12.4–40.3)
ORR (CR+PR)	37 (78.7)	(64.3–89.3)	28 (68.3)	(51.9–81.9)
SD	2 (4.3)	(0.5–14.5)	4 (9.8)	(2.7–23.1)
PD	4 (8.5)	(2.4–20.4)	4 (9.8)	(2.7–23.1)
Not evaluable	0	(0.0–7.5)	1 (2.4)	(0.1–12.9)
Not assessed	4 (8.5)	—	3 (7.3)	—
Missing	0	—	1 (2.4)	—

Abbreviations: CI=confidence interval; CR=complete response; FOLFOX=oxaliplatin, fluorouracil and leucovorin; IDMC=independent data monitoring committee; ORR=objective response rate; PD=progressive disease; RECIST=Response Evaluation Criteria in Solid Tumours; SD=stable disease.

**Table 3:** Grade 3–4 adverse events

	**FOLFOX4 (*N*=52)**	**5FU-cisplatin (*N*=43)**
	***n* (%)**	***n* (%)**
Number of patients with any grade 3 or 4 adverse events	39 (75.0)	31 (72.1)
*Haematological*	39 (75.0)	31 (72.1)
Neutropenia	12 (23.1)	9 (20.9)
Thrombocytopenia	5 (9.6)	5 (11.6)
Febrile neutropenia	5 (9.6)	2 (4.7)
Anaemia	2 (3.8)	3 (7.0)
Leucopenia	3 (5.8)	1 (2.3)
Febrile bone marrow aplasia	1 (1.9)	1 (2.3)
Pancytopenia	1 (1.9)	1 (2.3)
Dysphagia	10 (19.2)	7 (16.3)
Radiation-related oesophagitis	4 (5.8)	6 (14.0)
Stomatitis	3 (5.8)	0
Odynophagia	1 (1.9)	2 (4.7)
Oesophageal pain	1 (1.9)	1 (2.3)
Asthenia	4 (7.7)	4 (9.3)
Mucosal inflammation	4 (7.7)	1 (2.3)
Fatigue	2 (3.8)	2 (4.7)
Chest pain	0	2 (4.7)
Neutropenic infection	2 (3.8)	2 (4.7)
Weight decreased	2 (3.8)	2 (4.7)
Anorexia	2 (3.8)	2 (4.7)
Hypocalcaemia	0	2 (4.7)
